# Resource Usage and Performance Trade-offs for Machine Learning Models in Smart Environments

**DOI:** 10.3390/s20041176

**Published:** 2020-02-20

**Authors:** Davy Preuveneers, Ilias Tsingenopoulos, Wouter Joosen

**Affiliations:** imec–DistriNet, KU Leuven, Celestijnenlaan 200A, B-3001 Heverlee, Belgium; ilias.tsingenopoulos@cs.kuleuven.be (I.T.); wouter.joosen@cs.kuleuven.be (W.J.)

**Keywords:** resource optimization, hyperparameter tuning, machine learning, smart environments

## Abstract

The application of artificial intelligence enhances the ability of sensor and networking technologies to realize smart systems that sense, monitor and automatically control our everyday environments. Intelligent systems and applications often automate decisions based on the outcome of certain machine learning models. They collaborate at an ever increasing scale, ranging from smart homes and smart factories to smart cities. The best performing machine learning model, its architecture and parameters for a given task are ideally automatically determined through a hyperparameter tuning process. At the same time, edge computing is an emerging distributed computing paradigm that aims to bring computation and data storage closer to the location where they are needed to save network bandwidth or reduce the latency of requests. The challenge we address in this work is that hyperparameter tuning does not take into consideration resource trade-offs when selecting the best model for deployment in smart environments. The most accurate model might be prohibitively expensive to computationally evaluate on a resource constrained node at the edge of the network. We propose a multi-objective optimization solution to find acceptable trade-offs between model accuracy and resource consumption to enable the deployment of machine learning models in resource constrained smart environments. We demonstrate the feasibility of our approach by means of an anomaly detection use case. Additionally, we evaluate the extent that transfer learning techniques can be applied to reduce the amount of training required by reusing previous models, parameters and trade-off points from similar settings.

## 1. Introduction

Recent technological advancements in software and hardware have enabled the realization of context-aware applications in intelligent environments. Internet of Things (IoT) applications are processing growing amounts of sensor information to monitor everyday environments. Typical application examples include those supporting the elderly in smart homes [1], the ones monitoring environmental parameters in smart cities [2], smart health applications on wearable devices [3] and fault detection solutions for smart manufacturing [4].

Smart applications tap into a wealth of information derived from raw data by sophisticated data analytics techniques, including artificial intelligence methods and machine learning algorithms. While traditional machine learning has been applied successfully in many areas, we are now witnessing the adoption of deep learning methods and models proliferating. The main reason is the ability to recognize and extract complex patterns without the need to manually craft complex high-level features—upon which traditional machine learning methods usually depend— from raw data. Deep learning is now also finding its way into a variety of smart applications [5,6,7,8,9]. However, as these techniques rely on vast amounts of data to train models, and as the evaluation of such models can be computationally expensive, applications are leveraging the cloud and use Machine Learning as a Service (MLaaS) [10,11] to extract valuable information and make predictions in a scalable manner. The advantage of MLaaS in a resource rich deployment environment is that it simplifies and accelerates hyperparameter tuning [12]. Hyperparameter tuning or optimization is the process of automatically testing different configurations for training a machine learning model. Contrary to model parameters that are learned or estimated from data to make predictions, a hyperparameter is a parameter whose value is set before starting the model training process. Typical examples of hyperparameters for a variety of machine learning methods are the number of leaves or the depth of a decision tree, the number of clusters in k-means clustering, the number of estimators in a random forest, the number of hidden layers or the learning rate for a deep neural network, etc. Tuning the hyperparameters of a machine learning model can be done through simple grid search or random search strategies, or through advanced methods such as Bayesian optimization or genetic algorithms [13,14].

Edge computing [15] is a mainstream distributed computing paradigm that aims to bring computation and data storage closer to the location where they are needed in order to save network bandwidth or reduce the latency of application requests. For smart environments, the consequence is that the evaluation of machine learning models is also being shifted towards edge devices. A typical use case is federated machine learning [16,17,18], a machine learning technique that trains a shared prediction model across multiple decentralized nodes. These train the model with their own local data samples and do not exchange their data samples with other nodes. The technique was used amongst others on smartphones [19] to enhance the next-word prediction for virtual keyboards. As training data never leaves the node, federated learning was previously conceived as a means to maintain the confidentiality of training data. However, recent research has shown that this assumption may not always hold [20,21].

The challenge that we address in this research is two-fold: First, the case is that most hyperparameter tuning frameworks typically optimize the hyperparameters with respect to a single model criterion—usually the error or misclassification rate—to obtain the model with the best performance. However, for edge devices with resource constraints, such as sensors, gateways, wearables, etc., the most accurate prediction model may be too memory or battery demanding, too large to fit on a CUDA accelerated deep learning device (e.g., NVIDIA Jetson TX2 embedded system) or too computationally complex to be evaluated on the edge device, especially for applications where inference times faces stringent response latency constraints. Secondly, it is resource and time expensive to thoroughly tune hyperparameters, a process that results in knowledge about the specific use-case and deployed model that is discarded on the next iteration. Operating under significant resource and time constraints necessitates the use of the information already accumulated on optimal architecture and hyperparameter choices.

**Figure 1:**
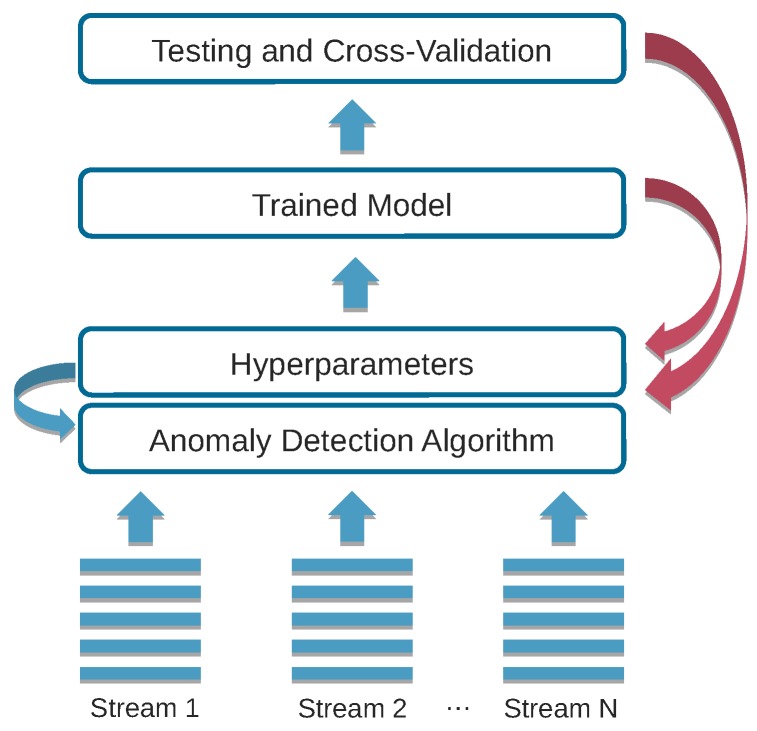
Hyperparameter tuning with resource trade-offs for an anomaly detection algorithm.

We present a hyperparameter tuning framework that also considers resource trade-offs when selecting the best model for deployment in smart environments, as depicted in Figure 1. Our contribution leverages multi-objective optimization [22] to find acceptable trade-offs between model accuracy and resource consumption. Our framework implements a two-stage approach. The first stage explores the high-dimensional search space in a resource rich environment, such as a high-end workstation or server with many CPUs and a large amount of memory. This multi-objective optimization process results in a collection of models of which some are on the Pareto front (Models on the Pareto front present the trade-offs between the optimization objectives and are considered equally good). Models that are not on the Pareto front have a counterpart on the Pareto front that is equal or better in terms of all the optimization objectives. The second stage re-evaluates the found models on the target device for an accurate characterization of the resource consumption. We then analyze how well the set of ‘best’ models on the Pareto front—as well as the hyperparameters of these models—transfer to resource constrained target devices. We investigate this in relative terms by comparing the models on the Pareto fronts on both test environments, and in absolute terms by analyzing the memory and CPU usage impact on the target device. Carrying out the hyperparameter tuning directly on the target device is often not feasible due to the resource limitations of the target device. The hyperparameter tuning process imposes high memory requirements for evaluating multiple models on large training sets. It already takes several hours on a high-end server or workstation, even when evaluating multiple models in parallel. Furthermore, re-evaluating the obtained models standalone on the target device is necessary to get an accurate resource usage without the memory overhead of the hyperparameter framework. Indeed, in a production environment, one would only deploy the best model on the target device and not the actual hyperparameter tuning framework. We demonstrate the feasibility of our approach by means of an anomaly detection [23] use case. As computing the Pareto front for optimal solutions is time consuming, even on a workstation or server, we also evaluate to what extent transfer learning techniques [24,25] can be applied to reduce the amount of training required by reusing previous models, parameters and trade-off points from similar settings [26,27]. Contrary to other works that pursue a resource-individual approach, our framework offers a single integrated solution for hyperparameter tuning with multiple resource trade-offs. The main contributions of this work can be summarized as follows:

- Hyperparameter tuning with resource trade-offs on top of existing traditional machine learning and deep learning frameworks.
- Practical feasibility analysis of transfer learning to speed up the computation of optimal configurations in similar settings.
- Evaluation of an anomaly detection use case involving network intrusion detection with attacks representing the anomalies, as well as other datasets.

These contributions were developed within the frame of the ICON RADIANCE research project (https://www.imec-int.com/en/what-we-offer/research-portfolio/radiance). In this project, we have the ambition to extend machine learning algorithms to detect anomalies in environments such as IT and telecommunication networks, enabling them to detect anomalies in real-time under resource constraints and adapt to changing contexts. This will cut training time and reduce the need for human involvement.

The remainder of this paper is structured as follows. In Section 2, we describe relevant related work on hyperparameter tuning and optimization. Section 3 describes our multi-objective optimization solution for finding acceptable machine learning models in terms of model accuracy and resource usage. In Section 4 we evaluate our framework on the ability to find Pareto-optimal solutions, measure the impact of transfer learning, and compare both scenarios against a baseline not taking resource constraints into consideration. We conclude in Section 5 summarizing the main insights and offering suggestions for further work.

## 2. Related Work

The hyperparameters of a machine learning method are parameters whose values are set prior to the start of the learning process. By contrast, the values of typical model parameters like neuron weights are computed during model training. Hyperparameter tuning is the problem of choosing a set of optimal hyperparameters for the learning problem. Recently, a variety of automated machine learning (AutoML) frameworks for traditional and deep learning methods has been proposed to tackle this challenge.

Auto-WEKA [28] is a Java-based AutoML framework that combines algorithm selection and hyperparameter optimization for the open source WEKA machine learning library [29]. Contrary to other frameworks that focus on deep learning only, Auto-WEKA explores the many learning and feature selection algorithms implemented in WEKA, resulting in an high-dimensional search space to search for the best performing configurations. It was one of the first frameworks to fully automate the process with Bayesian optimization, by utilizing Sequential Model-Based Optimization (SMBO) [30]. An instantiation of this optimization algorithm upon which Auto-WEKA depends was implemented by the Sequential Model-Based Algorithm Configuration (SMAC) tool [30]. For Python-based applications, Scikit-learn (https://scikit-learn.org/) is a popular and well-known framework for traditional machine learning. The AutoSklearn [31] framework follows the same AutoML approach first introduced in Auto-WEKA, combining a highly parametric machine learning library with a Bayesian optimization method. For Keras-based deep learning models, AutoKeras [32] similarly relies on Bayesian optimization to find the best neural network architecture.

Google Vizier [33] is the de facto black-box optimization and parameter tuning engine at Google, and underpins Google’s Cloud Machine Learning HyperTune (https://cloud.google.com/ml-engine/docs/hyperparameter-tuning-overview) subsystem. The advantage of Vizier and HyperTune over frameworks like Auto-WEKA is that the solution is offered as a service and minimizes the managerial overhead. Open source implementations of Vizier are available as well (e.g., Advisor, https://github.com/tobegit3hub/advisor). Vizier supports a form of transfer learning which leverages data from previous tuning experiments to accelerate the current one.

Multi-Objective Neural Architectural Search (MONAS) [34] is a hyperparameter optimization framework that aims to find neural network architectures optimized not only for prediction accuracy, but also other indicators. While the authors mainly focused on energy consumption as part of their evaluation and comparison with other approaches, the MONAS framework is extensible and can incorporate other optimization constraints. The multi-objective optimization process is based on reinforcement learning, using accuracy and energy consumption as components of the reward signal. The framework is also capable of enforcing hard constraints, such as a maximum peak power or minimum accuracy. DPP-Net [35] is another approach of device-aware search for Pareto-optimal neural network architectures. Similar to MONAS, it optimizes device-related (e.g., memory usage) and device-agnostic (e.g., accuracy or model size) objectives. Both approaches were evaluated in [36], showing that both frameworks are effective and are able to achieves Pareto-optimality with respect to the given objectives. While both resource-aware optimization frameworks are closely related to the objectives of our research, an open source implementation for MONAS and DPP-Net was not available for evaluation and adaptation purposes. Google’s AutoML uses reinforcement learning with gradient policy upgrade on top of Tensorflow to design the best neural network architecture. More recently, Google researchers proposed MnasNet [37], a solution that also incorporates the inference speed information into the main reward function of the search algorithm to find good trade-offs.

There is a multitude of software frameworks available that can assist in tuning the hyperparameters of a machine learning model. Table 1 contains an overview of automated hyperparameter optimization and tuning solutions. This list is not meant to be exhaustive, for a more comprehensive list of tuning solutions the reader can refer to: https://github.com/windmaple/awesome-AutoML and at https://github.com/markdtw/awesome-architecture-search.

The opportunity to accelerate hyperparameter tuning through transfer learning was explored in [26,27]. Transfer learning is a methodology where a model developed for a specific task is reused as the starting point for a model on another, related task. Yogatama et al. [26] proposed an algorithm for automatic hyperparameter tuning that generalizes across different datasets. It is an instance of Sequential Model-Based Optimization (SMBO) that uses deviations from the per-dataset means to transfer information. Perrone et al. [27] proposed a multi-task adaptive Bayesian linear regression model for transfer learning in Bayesian optimization. The challenge that they address is the fact that Bayesian optimization has an algorithmic complexity that is cubic in the number of evaluations. Their technique has a linear complexity to the number of function evaluations. It uses one Bayesian linear regression model per optimization problem, and then realizes transfer learning in a scalable way by coupling the models through a shared deep neural network.

Managing computational resources is also a key challenge for applications at the intersection of edge, cloud and services computing research, such as Multi-Access Edge Computing (MEC) [38]. A MEC system is an operator-owned system to run third party applications in a 5G landscape. Zanzi et al. [39] presented M${}^{2}$EC, an orchestration solution that acts as a MEC broker and exposes administration and management capabilities to MEC tenants. M${}^{2}$EC optimally allocates requested resources in compliance with the Service Level Agreements (SLA) of the tenants. Baresi et al. [40] proposed PAPS, a framework for the partitioning, allocation, placement and scaling of large-scale edge topologies and the decentralized self-management of containers and edge infrastructure. PAPS splits an edge topology into smaller communities that each elect a leader that is responsible for placing and allocating containers to cope with agreed SLAs for the incoming workload. These edge computing frameworks complement our research in that the resource usage characterization of the machine learning models provided by our solution are a perfect guide to defining these SLAs, while offering the necessary flexibility to edge resource management frameworks by allowing to trade one resource for another.

The gap that we aim to bridge is that existing solutions [33,41] focus primarily on finding the parameterization that achieves the best classification accuracy. A small subset of them consider resource usage and real-time detection as additional trade-offs to find the best parameter set for a particular model within a specific setting, configuration or deployment. Those are purposed to find solely neural network architectures, whereas equally effective traditional machine learning methods may exist. Furthermore, the practical feasibility of transfer learning [26,27] in the presence of multiple optimization objectives is not yet thoroughly explored.

## 3. Multi-Objective Optimization Approach

In this section, we describe the different steps behind our multi-objective optimization approach, as well as the datasets we have employed for our experimental evaluation. We explore both traditional machine learning techniques as well as deep learning methods:

1. We evaluate our framework on different types of anomaly datasets, including synthetic anomaly datasets as well as datasets collected in the real-world.
2. We train and test various machine learning models, and compare their performance as well as their resource usage (memory and CPU):
  - Binary classification vs. One-class classification.
  - Traditional machine learning vs. Deep learning methods.
3. We formally evaluate how adequately the Pareto front of optimal configurations transfers to the target device, in relative and absolute terms.

Binary classification techniques train models with normal and anomalous samples in the training set, whereas one-class classification techniques (such as One-Class SVM and Autoencoders) train only on normal samples. One-class classification methods learn a model of normal samples, and classify a test sample as anomalous when it deviates beyond a given threshold. As such, they can also identify unknown anomalies, whereas binary classifiers typically find anomalies only in respect to the samples that are part of the training set.

### 3.1. Anomaly Detection Datasets and Generation Tools

**Figure 2:**
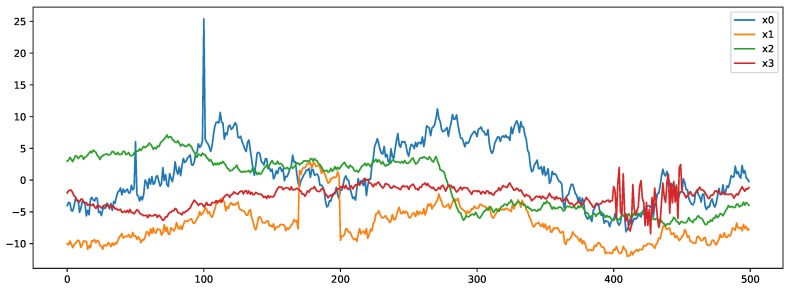
Synthetic anomaly data stream created with Anomaly Generator on Time Series (Agots).

As a test bed we are focusing on anomaly detection [23] use cases, as they cover a broad variety of applications ranging from fault detection or other exceptional situations in manufacturing scenarios up to security breaches manifesting themselves as deviations from normal user interaction or network traffic behavior. Key challenges with anomaly detection are (1) the number of classes are highly imbalanced, i.e., the anomalies are underrepresented compared to the normal samples, (2) new anomaly patterns may emerge that were not yet known when training the model and (3) the discovery of anomalies in one or more streams of data samples can be subject to real-time detection goals. Beyond real world anomaly datasets, the availability of software tools to generate synthetic datasets with known ground truth makes the systematic comparison for growing amounts of datasets more straightforward. The datasets we considered in our research are the following:

- **Yahoo! Webscope S5 Dataset**: Yahoo! Research [42] announced a benchmark dataset for time series anomaly detection. They have developed new anomaly detection algorithms causing fewer false positives to help service engineers looking to improve user experience and security. In order to evaluate the proposed algorithms, they created a large dataset that they have made public via their Webscope data-sharing program. The dataset includes real traffic to a Yahoo service, and incorporates also some synthetic data. There are 367 time series in the dataset. Each time series contains between 741 and 1680 observations recorded at regular intervals. They are also accompanied by an indicator series where a “1“ defines that the observation was an anomaly, and “0” indicator highlights normal traffic. The distinction between the real and synthetic data is the way the anomalies were determined. For the real data, these anomalies were determined by human judgment. The anomalies in the synthetic data were generated algorithmically. The dataset is available at https://research.yahoo.com/news/announcing-benchmark-dataset-time-series-anomaly-detection.
- **Enterprise software system anomalies**: This dataset was collected between August 2014 and October 2015 by Huch et al. [43] in a real-world industrial setting—monitoring 20 instances of a complex enterprise application—to test the feasibility of machine learning-based anomaly detection at runtime. The dataset consists of 831 metrics in 1-minute time intervals (in total $7.5\times 10^{6}$ data points), and contains time series data about the operating system, database connections and transactions, memory and CPU usage and many other metrics. The dataset is available at https://www.kaggle.com/anomalydetectionml/rawdata. The fact that 20 instances of the same application are monitored, makes this dataset ideally suited to evaluate the performance impact of transfer learning.
- **CICIDS 2017**: CICIDS 2017 [44] is a network intrusion detection dataset—available for download at https://www.unb.ca/cic/datasets/ids-2017.html—with network monitoring data collected over 5 days and from multiple machines. During the data gathering process, a variety of attacks—including Brute Force FTP, Brute Force SSH, DoS, Heartbleed, Web Attack, Infiltration, Botnet and DDoS— were carried out on specific days. In this work, we consider these attacks as anomalies. The raw data—i.e., bidirectional network traffic—was analyzed with a tool CICFlowMeter (http://www.netflowmeter.ca/netflowmeter.html) to produce 83 high-level statistical feature vectors.
- **Agots**: Anomaly Generator on Time Series (Agots) is an anomaly generation tool to produce completely synthetic multivariate data streams with the option to define (1) the number of values in each time series, (2) the number of time series and (3) the number of time series that should correlate. For the generation of outliers, the tool provides various parameters, including for the definition of extreme values, shifts, changing trends and variances. Figure 2 depicts an example with four time series in which two streams *x0* and *x1* are correlated, and where four types of outliers were added. These include two *extremes* in stream *x0* at offset 50 and 100, a *shift* in stream *x1* between offsets 170 and 200, a *trend* in stream *x2* between offsets 270 and 290 and a change in *variance* in stream *x3* between offsets 400 and 450. More details about Agots and an open source implementation can be found at https://github.com/KDD-OpenSource/agots. The dataset generated for our experiments contains 80 time series with 1500 data samples, and a variety of anomalies as depicted in Figure 2. The high-level feature vectors were computed over a window size of 10.

Another more sophisticated synthetic anomaly generation tool is AnoGen [45] developed by Facebook AI research. The approach relies on Variational Autoencoders (VAE) and Generative Adversarial Networks (GANs). AnoGen learns the normal and abnormal distributions using VAEs. To generate outliers or rare events in time series, AnoGen samples in the outlier region of the latent variable z. For datasets that only provide raw low-level time series data samples, we use tsfresh 0.13 (https://tsfresh.readthedocs.io) as a feature engineering tool in order to compute over 750 time and frequency based high-level features on sliding windows of different lengths.

### 3.2. Automated Benchmarking and Trade-Off Analysis

Here the objective is twofold: to identify anomalies in a time series dataset, and at the same time optimize resource consumption. Resource consumption can be measured both during the training phase, as well as during the testing phase. Evidently it will be different at both stages, given that for most machine learning methods the training phase is much more computationally and memory demanding. When we are concerned with purely statistical methods, there is no training time, only the successive update of the statistical indicators and thresholds.

**Figure 3:**
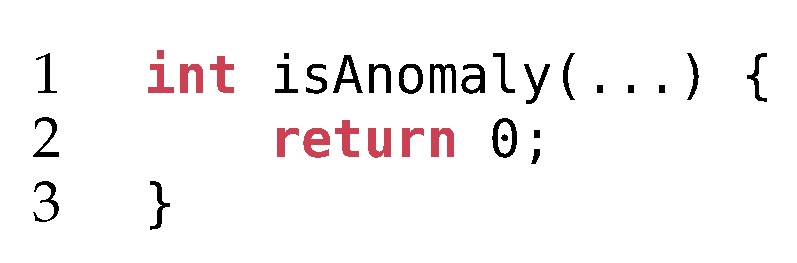
The least resource intensive anomaly detection algorithm.

By the very definition of anomaly, its occurrence is quite rare. When trading accuracy and resource consumption, it is very difficult to beat the algorithm illustrated in Figure 3. It depicts a straightforward anomaly detection algorithm which always returns “0“, interpreted as false or not an anomaly. From a systematic performance comparison point of view, it has several benefits:

- It requires no training.
- It is very low on resource consumption, both in terms of memory and processor usage.
- It is easily parallelizable and horizontally scalable.
- It has a pretty good accuracy (due to the imbalanced nature of the problem).

Obviously, the algorithm does not have any false positives (i.e., predicting an anomaly whereas in practice there is no anomaly), but it has a very high false negative rate (i.e., predicting no anomaly whereas in practice there is one). As a result, we have to be vigilant with identifying and positioning the performance metrics and trade-offs in relation to each other before drawing conclusions and generalizations. The end goal is therefore to produce a cost and trade-off model that would be able to take into account the following parameters and indicators:

- CPU time, wall clock time, memory vs. accuracy.
- Training vs. testing phase.
- Offline vs. online learning.

It follows then that a systematic analysis should vary one constraint at a time, and then tune the models with different parameters and configurations, e.g., fixing a memory budget which is a commonplace constraint in some deployment environments. Afterwards, the analysis can incorporate the effect of different wall clock times. Other practical considerations regarding the deployability of such machine learning models, are the feasibility of parallelization over multiple cores or nodes (e.g., for ensemble methods), the cost/benefit importance of feature engineering for specific application cases and the availability of hardware accelerated implementations.

#### 3.2.1. Binary Classification with Traditional Machine Learning Methods

In order to systematically compare between machine learning model families and configurations, we have built on top of AutoSklearn 0.6.0 and Java-based Auto-WEKA 2.6.1. By default these frameworks support only single objective optimization, as depicted in Appendix A.1 for the *errorRate* metric and the Yahoo dataset. The log trace in the appendix illustrates the configuration of the best machine learning model (*RandomForest*) found within 10 minutes, as well as the classification accuracy and the running time on the test set. We follow the same procedure for the other datasets, including the synthetic dataset generated by Agots. In the 10-minute exploration of the search space, a similar RandomForest model offers the best accuracy albeit with a slightly different attribute selection step (see the code in Appendix A.2). Similar hyperparameter tuning experiments were carried out with the Python-based AutoSklearn 0.6.0 tuning framework on top of Scikit-learn 0.21.3 machine learning library, as shown in Appendix A.3. In practice, the previous hyperparameter tuning experiments would have to run for several hours in order to find the best approximately configuration. These examples serve merely as an illustration and in the evaluation section, we report the results of a more comprehensive exploration on larger datasets.

By default, both Auto-WEKA and AutoSklearn optimize for only one metric, such as the error rate or accuracy. MONAS and DPP-Net [36], on the other hand, are natural extensions that search and optimize for multiple device-agnostic and device-aware constraints, resulting in gradually better models for all optimization objectives. The outcome of this process are tuples of objective performances where we can select the ones that are Pareto-optimal, that is they are optimal at least in one of the objectives. The entirety of these points constitute the Pareto-front, which can facilitate in choosing the fitting configuration for the occasion.

**Figure 4:**
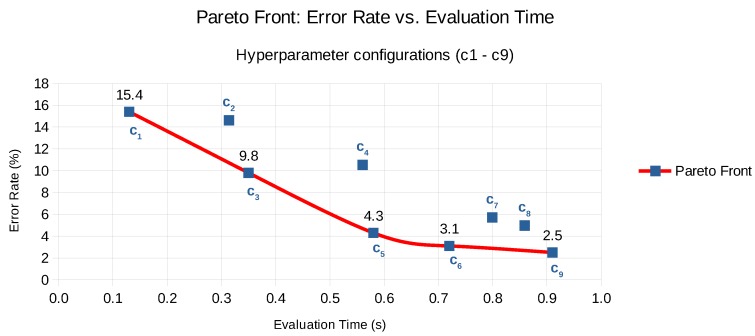
Pareto front of nine hyperparameter configurations ($c_{1}$ to $c_{9}$) demonstrating the error rate versus evaluation time trade-off.

Our approach takes the intermediate results of either Auto-WEKA or AutoSklearn, including the model itself as well as the performance metrics, to construct the Pareto front. This is illustrated for one of the hyperparameter experiments with AutoSklearn in Figure 4, depicting the Pareto front in red—including configurations $c_{1}$, $c_{3}$, $c_{5}$, $c_{6}$ and $c_{9}$ and a selection of the sub-optimal configurations in green—i.e., $c_{2}$, $c_{4}$, $c_{7}$ and $c_{8}$—that have either a higher error rate or are slower.

The main motivation for this approach is two-fold. First, it builds on top of existing frameworks without the need to modify code. Second, the search space for just a single optimization metric (e.g., *errorRate*) is already huge. Not only do Auto-WEKA and AutoSklearn need to compare the impact of different hyperparameters, they also have to do that across multiple feature selection and machine learning methods. With recommended exploration times for a single experiment being more than 24 hours, taking into consideration additional optimization objectives will impact considerably the computational demands. We therefore use the intermediate validated models and process monitoring in order to calculate the Pareto front.

#### 3.2.2. One-Class Classification with Traditional Machine Learning Methods

Contrary to binary classification techniques that train models using normal and anomalous samples in the training set, one-class classification methods learn a model using normal training samples only. A test sample is considered anomalous when it deviates too far from what has been modeled as the normal distribution. Traditional machine learning methods that fall into this One-Class Classification (OCC) category include One-Class K-means, One-Class K-nearest neighbors, One-Class SVM or a combination of these methods [46]. The first two methods also rely on thresholds to decide whether a new sample is anomalous or not, whereas One-Class SVM relies on two hyperparameters γ and ν for which the optimal configuration can be found through a simple grid search.

While traditional machine learning frameworks like Scikit-learn provide implementations for anomaly detection—such as One-Class SVM, Isolation Forest and Local Outlier Factor—the corresponding automated hyperparameter tuning frameworks are not befitting for this sort of unsupervised learning problems. AutoSklearn needs a loss function to tune the hyperparameters [47] and such a loss function is not provided for outlier detection by the underlying Scikit-learn framework. As a workaround for this limitation we use a custom grid search hyperparameter tuning solution, rather than AutoSklearn, to analyze the accuracy and resource usage trade-offs for this subset of unsupervised machine learning algorithms provided by Scikit-learn.

#### 3.2.3. One-Class Classification with Deep Learning-Based Methods

**Figure 5:**
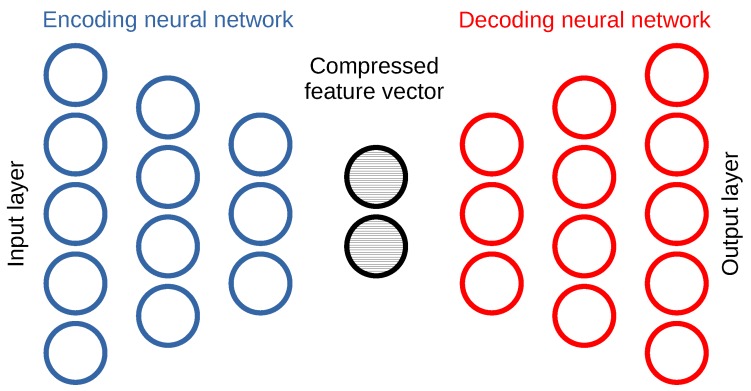
Deep autoencoder learning a compressed representation of normal input samples.

Moving to deep learning based anomaly detection methods, our investigation focuses mainly on autoencoder [48,49] neural networks. Autoencoders learn a compressed representation or an encoding of a normal dataset containing no anomalies, in an unsupervised manner. A typical autoencoder architecture is illustrated in Figure 5. We define these autoencoders using the Tensorflow 2.0 framework (https://www.tensorflow.org).

For an autoencoder that has sufficient learning capacity and has converged, the output sample closely resembles the input sample, as the decoder network learns how to reconstruct the original input from the compressed feature vector. At time of inference, the reconstruction error is used as a measure of how faithful the decoder’s reconstruction of the input is. Normal input samples have a low reconstruction error, whereas anomalous samples result in a high reconstruction error. A threshold in reconstruction error differentiates between the categories the input sample is classified, and this threshold can be set as the maximum reconstruction error observed during the training phase on normal samples. The hyperparameter search space for autoencoder models includes the different number of hidden layers, the number of neurons per layer, the number of layers in the encoder and the decoder, the learning rate and others.

Other opportunities to trade accuracy and resource usage is the use of the 16-bit half precision floating point format to represent the weights of the neural network rather than the standard 32-bit single precision floating point numbers. Not only does this reduce the memory consumption by half, it also leads to significantly faster network evaluations, especially on dedicated hardware such as mobile GPUs, but at the expense of slightly reduced accuracy. The latter is a approach often employed for Android or iOS mobile devices by converting regular Tensorflow models into Tensorflow Lite (TFLite) (https://www.tensorflow.org/lite) models for on-device inference. In fact, further resource usage improvements can be achieved through quantization of the models into 8-bit integers. The best performance is achieved on mobile devices with dedicated hardware and software (e.g., an Android device implementing the Neural Networks API on top of a hardware accelerator). For evaluating the converted models on workstations with a regular CPU, TFLite offers only an interpreter. Although more lightweight in terms of memory usage, the TFLite interpreter is not equally optimized for speed compared to the regular Tensorflow engine, and as such does not offer an accurate characterization of the speed of network evaluation. That is why this Tensorflow model representation trade-off is not explored within the hyperparameter tuning and trade-off analysis process.

**Figure 6:**
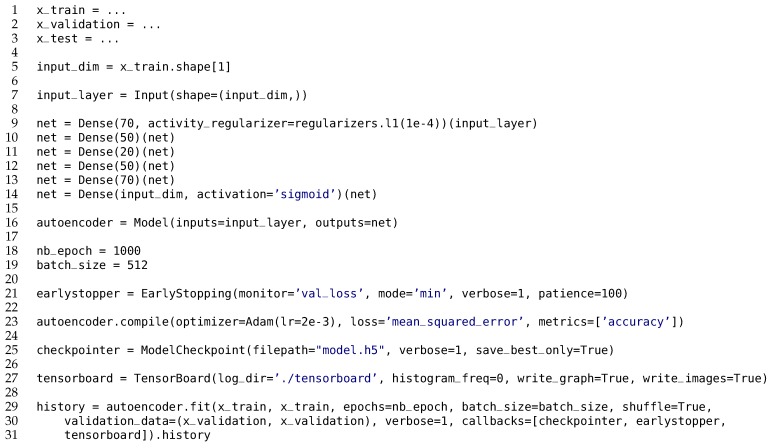
Autoencoder in Tensorflow 2.0 for the CICIDS 2017 dataset.

One of the main reasons to focus exclusively on this kind of neural networks is that for training they require data samples representative of normal behavior only. In an open-world assumption this property makes them more practical compared to classification models that can only recognize anomaly patterns only if they were previously trained on them. Autoencoders can be used on raw data inputs as well as high-level feature vectors. This is illustrated for the CICIDS 2017 dataset in Figure 6 using the high-level feature vectors as input for the autoencoder. The high-level features were computed by the CICFlowMeter tool.

Using the CICIDS2017 dataset as an example, from an input feature vector of 78 statistical features, the autoencoder constructs a compressed feature vector of size 20 through a series of densely connected layers before reconstructing it back into its original representation. The neural network is trained by minimizing the reconstruction loss and its convergence is evaluated on the validation dataset. The reconstruction loss defined as the mean squared error between the autoencoder’s output feature vector and the expected output feature vector, which is the same the original input feature vector. The network will be trained for at most 1000 epochs with a batch size of 512, and training will stop early when no improvement on the validation loss is achieved in the last 100 epochs.

### 3.3. Transferability of Pareto Fronts to Different Contexts

**Figure 7:**
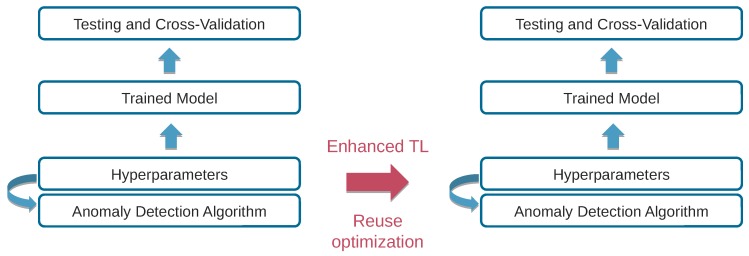
Transfer learning to speed up hyperparameter tuning and training.

Optimal model selection and hyperparameter optimization is an indispensable and time-consuming process in finding the highest performance model for the given task. When we take resource usage into consideration, it follows that identifying the optimal trade-off points becomes a challenging, non-trivial task. For that purpose we investigate to what extent transfer learning methodologies [24,25,26] are applicable in this domain in order to reduce the amount of training required by reusing previous model configurations, parameters and trade-off points from similar settings in order to bootstrap and inform future exploration. The overall approach is depicted in Figure 7.

Contrary to previous approaches that consider only a single optimization objective, such as the misclassification or error rate, moving towards multi-objective optimization we are compelled to account for the resource usage characteristics, including CPU time and memory consumption. In practice a straightforward method to accomplish this is to investigate whether the Pareto front of hyperparameters of a previous tuning experiment remains similar to the new one. The new tuning experiment may differ in two ways:

- A similar dataset collected in a different environment.
- A different deployment and evaluation environment (e.g., with a CUDA hardware accelerator).

To investigate the feasibility of the approach, we will employ the CICIDS 2017 network intrusion detection and the Enterprise software system anomalies datasets. By tuning the hyperparameters and learning a model on a subset of the machines in the network—possibly in a federated learning manner too—we can verify whether the model is equally effective—i.e., also with regard to the Pareto front—for the other machines and configurations in the network.

The methodology we follow re-evaluates the various hyperparameter configurations in the new context based on a distance metric from the original Pareto front (see Figure 4), and demonstrates the amount of time saved compared to executing a full exploration phase from the start.

## 4. Evaluation

In this section we assess and demonstrate the multi-objective hyperparameter tuning process for traditional machine learning and deep learning methods along with the computation of the Pareto fronts. Additionally, we evaluate the effectiveness of transfer learning in minimizing the amount of time it requires to optimally tune the hyperparameters in comparable settings.

In principle, the effectiveness of a model can be measured by means of the accuracy metric, defined in terms of true positives (TP), true negatives (TN), false positives (FP) and false negatives (FN):

(1) $$Accuracy=\frac{TP+TN}{TP+TN+FP+FN}$$

However, anomalies are usually underrepresented in the test set compared to the normal samples. A classifier that is unable to find any anomalies, such as the one in Figure 3, will have a high accuracy. Indeed, TP and FP will be equal to 0, such that the accuracy in this particular case becomes Accuracy=TN/(TN+FN). When the number of anomalies in the test set is relatively small, the value of FN will be small too, resulting in a high accuracy. That is why we must either ensure that all classes (i.e., normal and anomalous samples) are equally balanced in the test set, or use a different metric that can deal with class imbalance, such as the F1 score defined as the harmonic mean of the precision and recall:

(2) $$Precision=\frac{TP}{TP+FP}$$

(3) $$Recall=\frac{TP}{TP+FN}$$

(4) $$F1=2*\frac{Precision*Recall}{Precision+Recall}=\frac{2*TP}{2*TP+FP+FN}$$

For the one-class classification methods, we will also be using operating characteristic curve (ROC) curves as a way to represent the balance between true positive rate and false positive rate of a binary classifier system as its discrimination threshold is varied, as well as F1 score as a metric for binary classification.

The base deployment environment for the hyperparameter tuning is a Dell PowerEdge R620 server with 64GB of memory and two Intel Xeon E5-2650 (8 cores) CPUs running at 2.00GHz and hyperthreading enabled (resulting in 32 virtual cores). The peak memory usage for any process is measured as the *maxrss* value reported by the *getrusage()* system call on Linux.

### 4.1. Traditional Machine Learning Trade-Offs

AutoSklearn uses up to 2 h to find the best machine learning model and set of hyperparameters. For the Yahoo anomaly benchmark dataset, we use *tsfresh* to compute high-level feature vectors on a sliding window of 11 elements. In the case the 11-element sequence contains at least one anomaly, we consider the whole sequence as anomalous. The combined dataset contains 89,935 samples, and we use 80% (or 71,948 samples) for training and validation (split according to a 67/33 ratio) in order find the best model and hyperparameters. The remaining 20% is used for testing and measuring resource usage. The dataset was split with stratified sampling such that the training, validation and test sets have approximately the same percentage of samples of each target class (i.e., normal and anomalous) as the original complete dataset.

**Figure 8:**
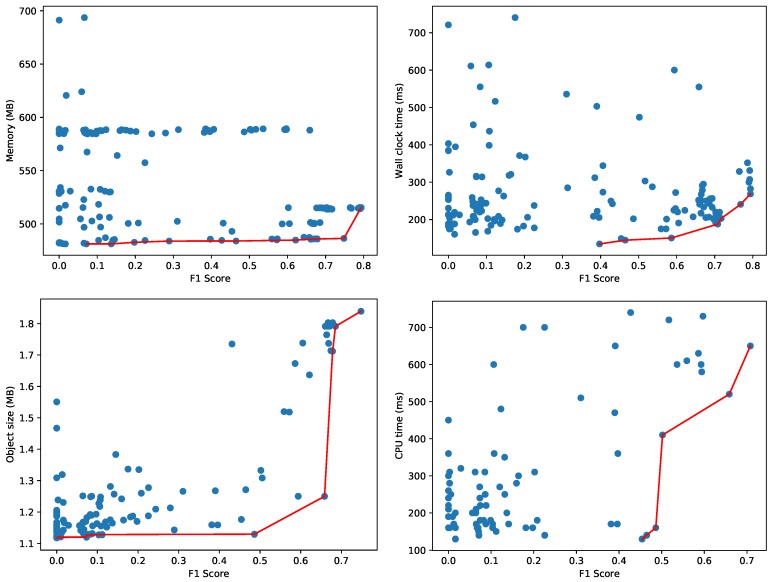
Pareto fronts for memory (**top left**), wall clock time (**top right**), object size (**bottom left**) and CPU time (**bottom right**) for the Yahoo! Webscope S5 Dataset.

AutoSklearn constructed 177 configurations of machine learning models and hyperparameters, trained until they converged and recorded the accuracy on the validation set. Figure 8 depicts the best machine learning models, evaluated on a test set—not overlapping with the training and validation set—to construct the Pareto fronts. We monitor the amount of memory used for the process and the wall clock time (i.e., the elapsed real time) for evaluating the test dataset. While the validation dataset may give a reasonable estimate for accuracy, the memory and CPU usage are more difficult to measure accurately during the hyperparameter tuning as multiple configurations are evaluated in parallel. That is why each configuration is measured sequentially on a test dataset as a separate process to obtain an accurate resource usage characterization. We apply a filtering, also in the reported figures, where configurations with an extremely high resource usage (i.e., more than 750 MB or taking more than 750 ms) are discarded. As an example, one configuration used 1140 MB and another required more than 8 s wall clock time (41 s CPU time) to evaluate the 17,987 samples in the test set. We also filtered out the results of a dummy classifier that AutoSklearn uses to set a baseline for the other models. This dummy classifier makes random predictions, and is therefore relatively fast:

- **Memory**: 448 MB.
- **Wall clock time and CPU time**: < 1 ms.

The memory consumption in Figure 8 provides a clear indication of the minimum memory usage for the whole Python process to load the test dataset and the necessary software libraries (e.g., AutoSklearn, Scikit-learn, Pandas, etc.). Figure 8 also depicts the Pareto fronts for the memory consumption of just the data object representing the machine learning model (i.e., only the size of the model object and not any software libraries, datasets or other objects). The majority of models use less than 2 MB as shown on the bottom left. As the wall clock time depends heavily on the effective use of the available cores on CPU, the figure also illustrates on the bottom right the Pareto front with respect to the actual CPU time used to evaluate the model on the test samples.

**Figure 9:**
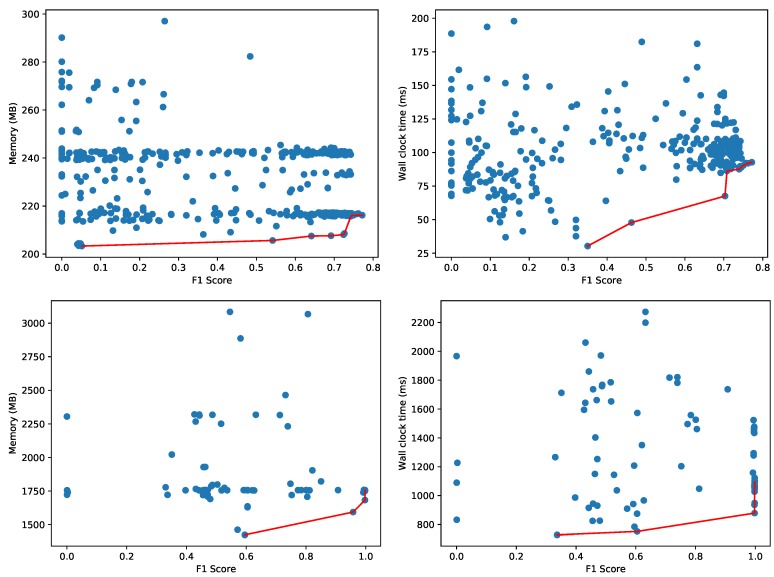
Pareto fronts for memory (**left**) and wall clock time (**right**) for the Agots synthetic dataset (**top**) and CICIDS 2017 dataset (**bottom**).

Figure 8 depicts two dimensional Pareto fronts. The Pareto front for multiple optimization objectives composes a multi-dimensional surface, but this surface is difficult to represent and interpret as-is when the objectives are more than 2. In the presence of hard constraints, this surface can still be projected to the two-dimensional space in order to facilitate the choice of the optimal model. We repeated the same experiment with the Agots synthetic dataset and plot the memory and wall clock time Pareto fronts in Figure 9. Compared to the Yahoo dataset in Figure 8, the overall memory consumption and evaluation times are significantly lower for the Agots dataset (4473 test samples), and significantly higher for the CICIDS 2017 dataset (566,149 test samples). This is according to our expectations, given the complexity of both datasets relative to the Yahoo dataset.

### 4.2. Traditional One-Class Classification Methods Versus Deep Learning-Based Autoencoders

We use the CICIDS 2017 dataset to compare the deep learning-based autoencoders with a selection of traditional one-class classification methods. These are all trained on normal samples and tested on a mix of normal and anomalous samples. As AutoSklearn is mainly intended for supervised classification problems and not this kind unsupervised learning methods, we implemented a simple grid search for the γ and ν hyperparameter of the One-Class SVM algorithm implemented in the Scikit-learn framework:

- γ: [0.001, 0.005, 0.01, 0.05, 0.1, 0.5, 1, 5, 10, 50, 100, 500].
- ν: [0.001, 0.003, 0.005, 0.008, 0.01, 0.03, 0.05, 0.08, 0.1, 0.3, 0.5, 0.8, 1].

**Figure 10:**
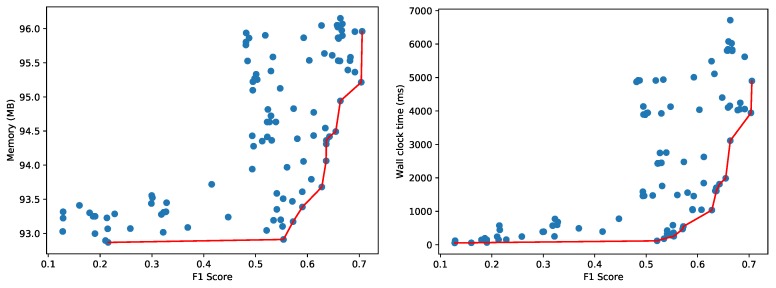
Pareto fronts for memory (left) and wall clock time (right) for the CICIDS 2017 dataset using the One-Class SVM classifier.

For these 156 hyperparameter combinations and training with 36,363 samples, Figure 10 depicts the memory usage and wall clock time to classify 13,318 test samples, of which 9091 normal and 2227 anomalous (same class distribution as the original dataset). Memory consumption for the whole Python process varied between 95 and 114 MB for the configurations on the Pareto front. The highest F1 score obtained is 0.706 with a test time of around 4.90 s for all 13,318 test samples (wall clock time and CPU time are equal as only one CPU core is used). Furthermore, the implementation of One-Class SVM is not multi-core enabled, so the classification of the 13,318 test samples only uses one of the 32 virtual cores of the Intel Xeon CPU.

These results are in sharp contrast with the ones provided by the autoencoders implemented in the Tensorflow 2.0 library. To set a baseline, merely loading and activating the Tensorflow library by creating a tensor holding one random floating point value causes the Python process to consume 242 MB.

The dataset is split in a training set of size 1,590,881 and a validation set of size 340,904, both only containing normal feature vectors. The test set is the same as for the One-Class SVM classifier, having a mix of normal and anomalous samples. We explore different neural network architectures, as illustrated in Figure 6 and explained in Section 3.2.2, but with varying numbers of layers and neurons per layer, etc.

One of those autoencoder configurations is depicted in Figure 6. The training of this particular neural network architecture ends at epoch 227. The lowest loss value achieved within these 227 epochs is $1.7e^{-4}$. The wall clock time to train this Tensorflow 2.0 model is 2705 s (CPU time is 5867 s), and the peak memory usage of the Python process during training is 4,046,200 kilobytes, or about 3951 megabytes. The size of the Tensorflow model when serialized to disk is just 303,600 bytes.

**Figure 11:**
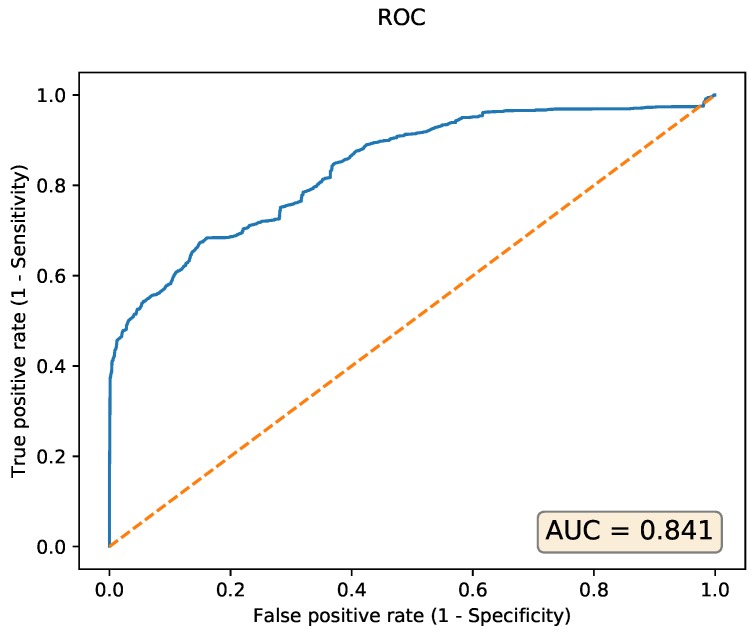
The area under the operating characteristic curve (ROC) curve for the CICIDS 2017 autoencoder with a 70-50-20-50-70 neurons per layer configuration.

The accuracy of the model in terms of the area under the receiver operating characteristic curve (ROC) curve is AUC = 0.841, as depicted in blue in Figure 11.

The false positive rate and true positive rate metrics are computed for different thresholds of the reconstruction error that classify input samples as either normal or anomalous. The dashed diagonal line depicts a random classifier that assigns to normal or anomalous classes each with probability 0.5, resulting in a AUC = 0.5. The evaluation of the 13,318 test samples takes 1.22 s wall clock time and 1.36 s CPU time. Peak memory usage is 341,228 kilobytes or 333 megabytes. We re-train the same autoencoder on an NVIDIA Titan V GPU with a 100 times larger batch size, i.e., 51,200 rather than 512. The training process now completes with a wall clock time of 338 s (versus 2705 s on the CPU).

**Figure 12:**
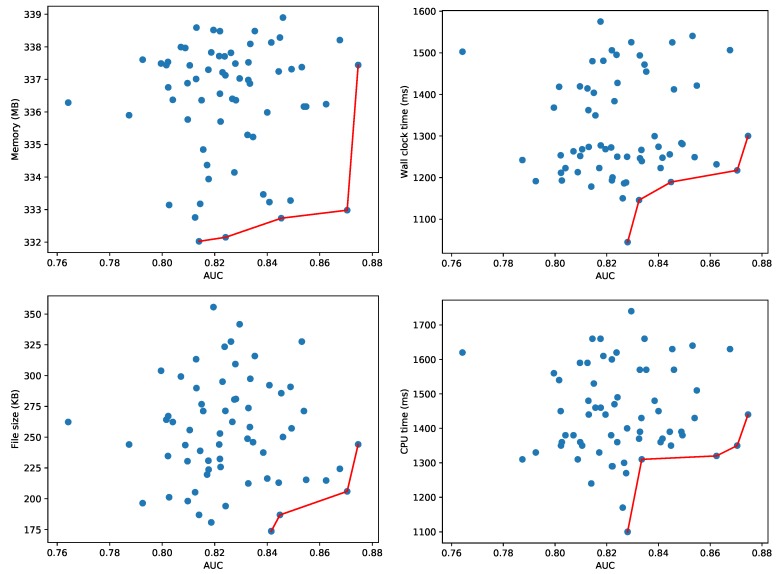
Pareto fronts for memory (**top left**), wall clock time (**top right**), file size (**bottom left**) and CPU time (**bottom right**) for the CICIDS 2017 dataset using 64 configurations of autoencoders.

Other autoencoders with different number of layers, neurons per layer, etc. will have variable performance metrics and resource usage characteristics, both for the training and testing phases. As the neural network training is significantly faster on the CUDA accelerated hardware, we explore other hyperparameter configurations on the Titan V GPU. However, for a fair comparison with the One-Class SVM method, we evaluate the 13,318 test samples for the found models on the CPU. Figure 12 depicts the Pareto-fronts for 64 different configurations of autoencoders. Contrary to the previous experiments where we analyzed the Pareto front using the F1 score of the different models, we now use the AUC metric to compare the different autoencoders. For an autoencoder, we need to set a threshold to categorize normal and anomalous samples. Only for a given threshold, we can compute the corresponding F1 score. We vary the threshold to compute the area under the ROC curve, and use this threshold-independent metric to compare the different autoencoders. An alternative is to fix a threshold for all 64 models, and compare the F1 scores for this given threshold. There is no significant difference in the peak memory usage for any of the autoencoder models, as they vary between 332 MB and 339 MB, a difference of about 2%. The wall clock time varies between 1000 ms and 1600 ms, whereas the CPU time varies between 1100 and 1750 ms (higher than the wall clock time due to the use of multiple CPU cores). This means One-Class SVM is more efficient in terms of resource usage.

### 4.3. Transferability of the Pareto Front to Similar Datasets: The Machine Learning Models

The construction of Pareto fronts is a procedure that leads to designs that are simultaneously optimal over multiple criteria. Populating a Pareto front is a computationally expensive task and in that regard a methodology to transfer previously optimal configurations in new but related contexts can prove invaluable. Despite the contextual proximity of a task, the effectiveness of transferring the Pareto optimal configurations should be evaluated in a consistent and representative manner. For that reason a hypervolume indicator [50] is preferred as a metric to measure the effectiveness. Specifically this hypervolume indicator is the volume contained by the convex hull constructed by the two Pareto fronts, the origin one and the target one. In this manner we can get a direct assessment, even though still relative, of the effectiveness of the transfer between different contexts.

The impact of transfer learning is tested on the Enterprise anomaly dataset. In the following experiments, we use the operational data that are already processed and labeled (https://www.kaggle.com/anomalydetectionml/features) rather than the raw data. This dataset contains 7,501,347 feature vectors for 10 different hosts (i.e., the first feature in each feature vector), and each feature vector contains 235 features, including whether the feature vector is normal or an anomaly. We split the data per host, and use the data of three hosts (i.e., *lphost11*, *lphost14* and *lphost15*) to train various models, and investigate how well the Pareto front of models transfers to three separate hosts (i.e., *lphost09*, *lphost10* and *lphost17*).

**Figure 13:**
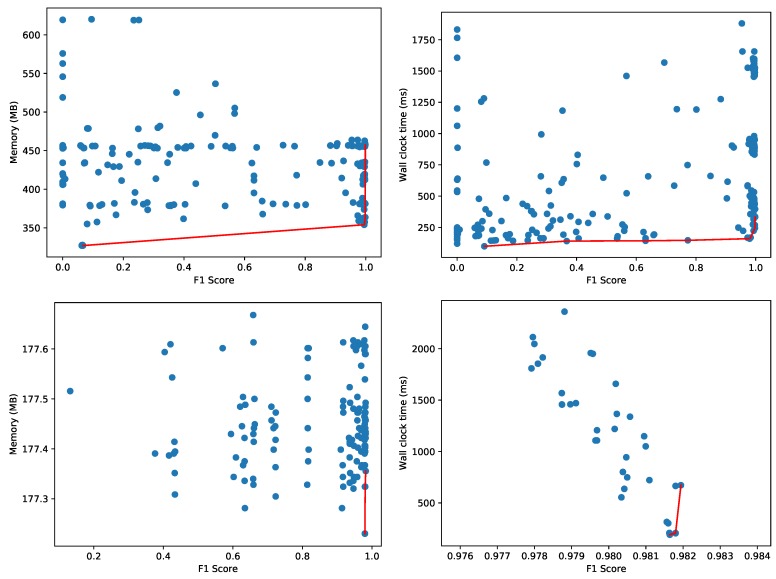
Pareto fronts for memory (**left**) and wall clock time (**right**) for the Enterprise anomaly dataset using AutoSklearn (**top**) and One-Class SVM (**bottom**).

**Figure 14:**
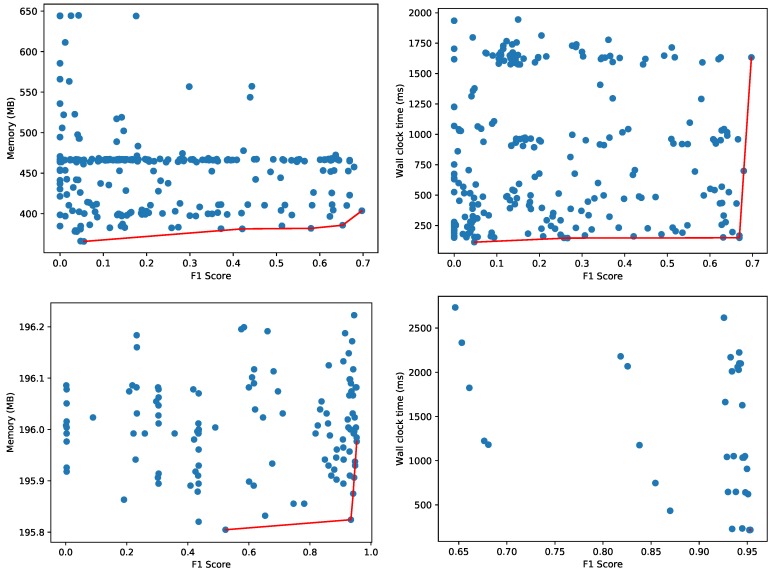
Pareto fronts for memory (**left**) and wall clock time (**right**) for the Enterprise anomaly dataset re-using AutoSklearn (**top**) and One-Class SVM (**bottom**) models for different hosts.

Figure 13 depicts the Pareto fronts for peak memory usage and wall clock time using AutoSklearn for the supervised machine learning scenario, and the One-Class SVM classifier for the unsupervised variant. These configurations are trained for the *lphost11*, *lphost14* and *lphost15* hosts. To measure the impact of transfer learning, we first evaluate to what extent the same trained models can be reused for the *lphost09*, *lphost10* and *lphost17* hosts. The results for the AutoSklearn models and One-Class SVM are depicted in Figure 14.

It is evident that the F1 score drops significantly for the binary classifiers found by AutoSklearn. Whereas the maximum value for the F1 score on the original data, as depicted in Figure 13, goes up to 0.998, the metric drops to 0.697 in Figure 14. The One-Class SVM models transfer better, with the F1 score dropping from 0.982 to just 0.953. Interestingly enough, the wall clock time Pareto front in Figure 14 collapses to two data points with a similar F1 score and wall clock time:

- Pareto-optimal model 1: F1 score = 0.9531404, Wall clock time: 216.9302 ms.
- Pareto-optimal model 2: F1 score = 0.9527573, Wall clock time: 215.0607 ms.

These results show that transferring trained models directly is not optimal and the performance drops are not consistent, as illustrated by the considerably low F1 score of 0.697. In the next section, we investigate the impact of transferring the hyperparameter configurations in a Pareto front in place of the machine learning models themselves.

### 4.4. Transferability of the Pareto front to Similar Datasets: The Hyperparameters

With this approach, we take the hyperparameters of the AutoSklearn models on the Pareto front for the origin dataset (depicted in Figure 13), and retrain model with the same configurations on the target dataset. In the memory experiment, from the 277 models there are five on the corresponding Pareto front (see Table 2), whereas eight models are on the wall clock Pareto front (see Table 3).

This experiment illustrates that even if the original model is not effective or accurate, one may train a new model in the new context with the same hyperparameters to obtain approximately the same results, depending on the proximity of the contexts. That is to say the computationally expensive hyperparameter tuning process can be replaced by retraining the models with the configurations on the original Pareto front, in this case from 277 models (training time = 7197 s) down to just 13 models (training time = 507 s).

### 4.5. Transferability of the Pareto front to a Different Target Device

In the final experiment, we evaluate the hyperparameter configurations situated on the Pareto front on a different evaluation environment. This new environment is an NVIDIA Jetson TX2 board, with a hex-core ARMv8 64-bit CPU and 8GB of memory.

**Figure 15:**
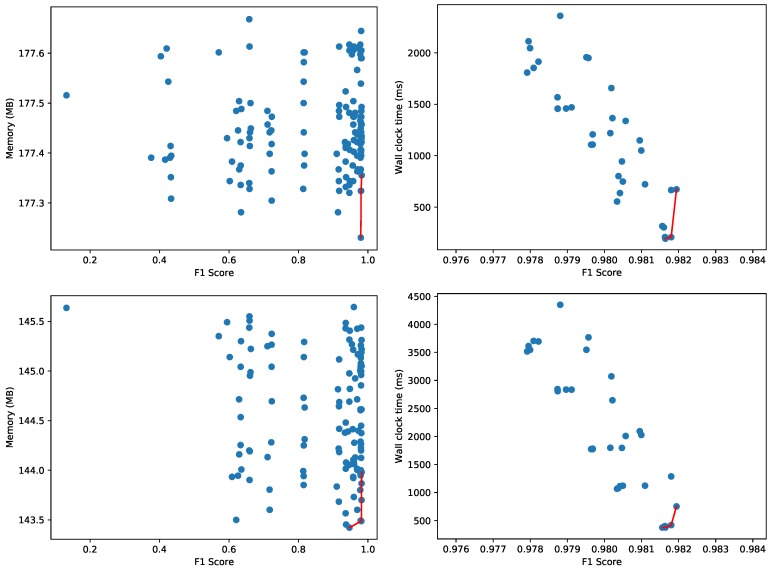
Pareto fronts for memory (**left**) and wall clock time (**right**) for the Enterprise anomaly dataset re-using One-Class SVM models on the Dell PowerEdge R620 server (**top**) and NVIDIA Jetson TX2 board (**bottom**).

As the test samples have remained the same, the accuracy of each hyperparameter configuration is also the same. The differences are in resource usage, i.e., the peak memory usage and the wall clock time to evaluate the test samples. As Figure 15 illustrates, the Pareto curve remains the same, but memory consumption is overall lower, whereas the wall clock time more or less doubled. This means that the transfer learning of the Pareto front is successful unless one aims to select a configuration that must meet strict resource limitations. In that case, it is sufficient to only re-evaluate the configurations on the Pareto front.

**Figure 16:**
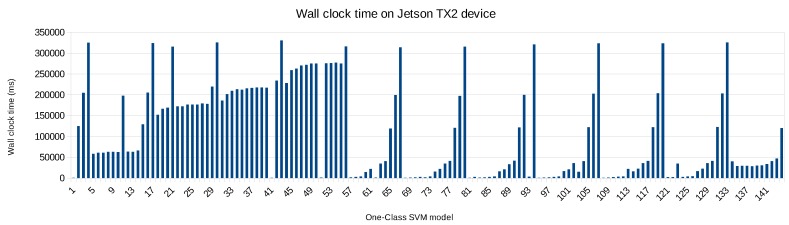
Wall clock time for all One-Class SVM models on the NVIDIA Jetson TX2 board.

Re-evaluating the 4 wall clock time Pareto-optimal hyperparameter configurations (initially obtained on the Dell PowerEdge R620 server but now testing again on the NVIDIA Jetson TX2 embedded board) takes 1922 ms, whereas fully exploring all 144 hyperparameter configurations on the embedded board takes 15,718,492 ms. The main reason is that some of these 144 models—filtered out from Figure 15—take more than 320,000 ms to test, as depicted in Figure 16. So the performance gain through transfer learning is significant, speeding up the evaluation with a factor ≈8000.

## 5. Conclusions

In this work we presented a comprehensive assessment and demonstration of the trade-offs between the performance of various machine learning models in resource critical environments. Following the proposed methodology we construct plots and Pareto-optimal surfaces over 4 datasets that can prove instrumental for the machine-learning engineer or practitioner in selecting the optimal model tailored to the desired task. Finally, we perform an extensive evaluation and highlight the trade-offs that demonstrate our methodology’s pertinence and applicability in resource-constrained environments.

Our experiments demonstrate that multi-objective hyperparameter optimization can be accelerated through transfer learning, but that not all machine learning methods and corresponding Pareto fronts transfer equally well. Furthermore, traditional machine learning methods can be more effective in terms of resource usage compared to deep learning-based neural networks. As much as that may sound as a predictable conclusion, in the near future the spread of new optimized hardware solutions—such as neuromorphic computing chips [51] that emulate the electrical behavior of neurons in the brain, or FPGA-based accelerators [52]—will enable the evaluation of neural networks at a very low energy cost and can turn into a tipping-point for deep learning at the edge.

As future work, we will investigate how alternatives to the One-Class SVM classification algorithm, such as the Isolation Forest method, affect the transferability of hyperparameters. Another exploration that should be considered is the computational cost of feature engineering for traditional machine learning methods compared to the intrinsic one carried out by deep learning models. To achieve a more efficient hardware resource utilization on typical mobile platforms and edge computing devices, we will investigate how our framework can be extended to measure additional indicators, such as the area usage, power consumption and latency. This would entail additional functionality that fully automates the hyperparameter tuning process, the deployment of machine learning models on the target device, as well as the evaluation of test data and the accurate measurement of these indicators. This would result in a more universal and comprehensive analysis of resource usage in relation to the performance of the deployed models.

## Figures and Tables

**Table 1:** Overview of hyperparameter optimization and tuning solutions.

Framework	URL
TPOT	https://epistasislab.github.io/tpot/
SMAC	https://github.com/automl/SMAC3
AutoWeka	http://www.cs.ubc.ca/labs/beta/Projects/autoweka/
AutoSklearn	https://automl.github.io/auto-sklearn/
AutoKeras	https://autokeras.com/
H2O AutoML	http://docs.h2o.ai/h2o/latest-stable/h2o-docs/automl.html
TransmogrifAI	https://transmogrif.ai
HyperAS	https://maxpumperla.com/hyperas/
DEvol	https://github.com/joeddav/devol
Talos	https://github.com/autonomio/talos

**Table 2:** Models on the wall clock Pareto front (columns with ${}^{*}$ report results for new test set).

Model ID	F1 Score	Memory (MB)	F1 Score ${}^{*}$	Memory (MB) ${}^{*}$
73	0.9983	457	0.9963	430
111	0.9980	381	0.9870	405
213	0.9980	363	0.9950	380
242	0.9960	357	0.9901	405
179	0.9947	354	0.9907	363

**Table 3:** Models on the wall clock Pareto front (columns with ${}^{*}$ report results for new test set).

Model ID	F1 Score	Wall Clock Time (ms)	F1 Score ${}^{*}$	Wall Clock Time (ms) ${}^{*}$
67	0.9983	334	0.9963	393
141	0.9973	244	0.9950	251
212	0.9930	223	0.9640	187
214	0.9830	169	0.9325	129
83	0.9797	160	0.9765	157
148	0.7726	147	0.8798	135
29	0.6309	143	0.6710	139
183	0.3686	140	0.2687	139

## Appendix A

### Appendix A.1. A Log Trace of Hyperparameter Optimization with Auto-WEKA for the Yahoo Dataset

1  Options: -seed 123 -timeLimit 10 -memLimit 8192 -nBestConfigs 10 -metric errorRate -parallelRuns 
 2
 3  === Classifier model (full training set) === 
 4
 5  best classifier: weka.classifiers.trees.RandomForest 
 6  arguments: [-I, 10, -K, 0, -depth, 0] 
 7  attribute search: null 
 8  attribute search arguments: [] 
 9  attribute evaluation: null 
10  attribute evaluation arguments: [] 
11  metric: errorRate 
12  estimated errorRate: 0.005789022298456261 
13  training time on evaluation dataset: 0.205 s 
14 
15  You can use the chosen classifier in your own code as follows: 
16
17  Classifier classifier = AbstractClassifier.forName("weka.classifiers.trees.RandomForest", new String[]{"-I", 
18    "10", "-K", "0", "-depth", "0"}); 
19  classifier.buildClassifier(instances); 
20 
21 
22  Correctly Classified Instances        4628               99.2281 % 
23  Incorrectly Classified Instances        36                0.7719 % 
24  Kappa statistic                          0.9846 
25  Mean absolute error                      0.084 
26  Root mean squared error                  0.1456 
27  Relative absolute error                 16.8096 % 
28  Root relative squared error             29.1268 % 
29  Total Number of Instances             4664 
30 
31  === Confusion Matrix === 
32            
33      a    b   <-- classified as 
34   2324    8 |    a = False 
35     28 2304 |    b = True 
36
37  === Detailed Accuracy By Class === 
38
39                 TP Rate  FP Rate  Precision  Recall   F-Measure  MCC      ROC Area  PRC Area  Class 
40                 0.997    0.012    0.988      0.997    0.992      0.985    1.000     1.000     False 
41                 0.988    0.003    0.997      0.988    0.992      0.985    1.000     1.000     True 
42  Weighted Avg.  0.992    0.008    0.992      0.992    0.992      0.985    1.000     1.000 
43           
44  Time taken to build model: 582.64 s 
45
46  Time taken to test model on training data: 0.28 s

### Appendix A.2. A Log Trace of Hyperparameter Optimization with Auto-WEKA for the Agots Synthetic Dataset

1  Options: -seed 123 -timeLimit 10 -memLimit 8192 -nBestConfigs 10 -metric errorRate -parallelRuns 
 2 
 3  === Classifier model (full training set) === 
 4
 5  best classifier: weka.classifiers.trees.RandomForest 
 6  arguments: [-I, 10, -K, 1, -depth, 0] 
 7  attribute search: weka.attributeSelection.BestFirst 
 8  attribute search arguments: [-D, 2, -N, 6] 
 9  attribute evaluation: weka.attributeSelection.CfsSubsetEval 
10  attribute evaluation arguments: [] 
11  metric: errorRate 
12  estimated errorRate: 0.007228915662650603 
13  training time on evaluation dataset: 0.025 s 
14
15  You can use the chosen classifier in your own code as follows: 
16
17  AttributeSelection as = new AttributeSelection(); 
18  ASSearch asSearch = ASSearch.forName("weka.attributeSelection.BestFirst", new String[]{"-D", "2", "-N", "6"}); 
19  as.setSearch(asSearch); 
20  ASEvaluation asEval = ASEvaluation.forName("weka.attributeSelection.CfsSubsetEval", new String[]{}); 
21  as.setEvaluator(asEval); 
22  as.SelectAttributes(instances); 
23  instances = as.reduceDimensionality(instances); 
24  Classifier classifier = AbstractClassifier.forName("weka.classifiers.trees.RandomForest", new String[]{"-I", 
25    "10", "-K", "1", "-depth", "0"}); 
26  classifier.buildClassifier(instances); 
27
28 
29  Correctly Classified Instances         824               99.2771 % 
30  Incorrectly Classified Instances         6                0.7229 % 
31  Kappa statistic                          0.9855 
32  Mean absolute error                      0.072 
33  Root mean squared error                  0.1298 
34  Relative absolute error                 14.4096 % 
35  Root relative squared error             25.9564 % 
36  Total Number of Instances              830 
37 
38  === Confusion Matrix === 
39
40     a   b   <-- classified as 
41   415   0 |   a = False 
42     6 409 |   b = True 
43
44  === Detailed Accuracy By Class === 
45
46                  TP Rate  FP Rate  Precision  Recall   F-Measure  MCC      ROC Area  PRC Area  Class 
47                  1.000    0.014    0.986      1.000    0.993      0.986    1.000     1.000     False 
48                  0.986    0.000    1.000      0.986    0.993      0.986    1.000     1.000     True 
49  Weighted Avg.   0.993    0.007    0.993      0.993    0.993      0.986    1.000     1.000 
50 
51  Time taken to build model: 550.29 s 
52
53  Time taken to test model on training data: 0.04 s

### Appendix A.3. A Log Trace of Hyperparameter Optimization with AutoSklearn for the Agots Synthetic Dataset

1  automl = autosklearn.classification.AutoSklearnClassifier( 
 2      time_left_for_this_task=600, 
 3      per_run_time_limit=60, 
 4      ml_memory_limit=8192, 
 5      n_jobs=2, 
 6      tmp_folder=’/tmp/autosklearn_agots_tmp’, 
 7      output_folder=’/tmp/autosklearn_agots_out’, 
 8      delete_tmp_folder_after_terminate=False, 
 9      delete_output_folder_after_terminate=False, 
10      disable_evaluator_output=False 
11  ) 
12
13  *** Statistics: 
14  auto-sklearn results: 
15    Dataset name: 83635e2c0977166dc0122ff29795dd92 
16    Metric: accuracy 
17    Best validation score: 0.890909 
18    Number of target algorithm runs: 100 
19    Number of successful target algorithm runs: 83 
20    Number of crashed target algorithm runs: 3 
21    Number of target algorithms that exceeded the time limit: 5 
22    Number of target algorithms that exceeded the memory limit: 9 
23
24  *** Models: 
25  [(0.200000, SimpleClassificationPipeline({’balancing:strategy’: ’weighting’, 
26    ’categorical_encoding:__choice__’: ’one_hot_encoding’, ’classifier:__choice__’: ’adaboost’, 
27    ’imputation:strategy’: ’median’, ’preprocessor:__choice__’: ’extra_trees_preproc_for_classification’, 
28    ’rescaling:__choice__’: ’none’, ’categorical_encoding:one_hot_encoding:use_minimum_fraction’: ’False’, 
29    ’classifier:adaboost:algorithm’: ’SAMME’, 
30    ’classifier:adaboost:learning_rate’: 0.290012331833807, 
31    ’classifier:adaboost:max_depth’: 1, 
32    ’classifier:adaboost:n_estimators’: 245, 
33    ’preprocessor:extra_trees_preproc_for_classification:bootstrap’: ’False’, 
34    ’preprocessor:extra_trees_preproc_for_classification:criterion’: ’entropy’, 
35    ’preprocessor:extra_trees_preproc_for_classification:max_depth’: ’None’, 
36    ’preprocessor:extra_trees_preproc_for_classification:max_features’: 0.5067944315035667, 
37    ’preprocessor:extra_trees_preproc_for_classification:max_leaf_nodes’: ’None’, 
38    ’preprocessor:extra_trees_preproc_for_classification:min_impurity_decrease’: 0.0, 
39    ’preprocessor:extra_trees_preproc_for_classification:min_samples_leaf’: 16, 
40    ’preprocessor:extra_trees_preproc_for_classification:min_samples_split’: 2, 
41    ’preprocessor:extra_trees_preproc_for_classification:min_weight_fraction_leaf’: 0.0, 
42    ’preprocessor:extra_trees_preproc_for_classification:n_estimators’: 100}, 
43  dataset_properties={ 
44    ’task’: 1, 
45    ’sparse’: False, 
46    ’multilabel’: False, 
47    ’multiclass’: False, 
48    ’target_type’: ’classification’, 
49    ’signed’: False})), 
50  ... 
51  ] 
52
53  *** Predict: 
54  Accuracy score 0.8313253012048193
